# Pathogenesis and prevention of placental and transplacental porcine reproductive and respiratory syndrome virus infection

**DOI:** 10.1186/1297-9716-44-95

**Published:** 2013-10-07

**Authors:** Uladzimir U Karniychuk, Hans J Nauwynck

**Affiliations:** 1 Laboratory of Virology, Faculty of Veterinary Medicine, Ghent University, Salisburylaan 133, 9820, Merelbeke, Belgium

## Abstract

Porcine reproductive and respiratory syndrome virus (PRRSV)-induced reproductive problems are characterized by embryonic death, late-term abortions, early farrowing and increase in number of dead and mummified fetuses, and weak-born piglets. The virus recovery from fetal tissues illustrates transplacental infection, but despite many studies on the subject, the means by which PRRSV spreads from mother to fetus and the exact pathophysiological basis of the virus-induced reproductive failure remain unexplained. Recent findings from our group indicate that the endometrium and placenta are involved in the PRRSV passage from mother to fetus and that virus replication in the endometrial/placental tissues can be the actual reason for fetal death. The main purpose of this review is to clarify the role that PRRSV replication and PRRSV-induced changes in the endometrium/placenta play in the pathogenesis of PRRSV-induced reproductive failure in pregnant sows. In addition, strategies to control placental and transplacental PRRSV infection are discussed.

## Table of contents

1. Morphology and function of the porcine placenta

2. Pathology of gestation in the swine

3. PRRSV infection in pregnant sows

3.1 Introduction

3.1 Clinical signs

3.1 Routes of PRRSV transmission

4. PRRSV infection in the conceptus

4.1 Embryo PRRSV infection during early gestation

4.1.1 Embryo PRRSV infection during early gestation upon intranasal sow inoculation

4.1.1 Embryo PRRSV infection during early gestation upon in utero inoculation

4.1 Fetal PRRSV infection during mid-gestation

4.1.1 Fetal PRRSV infection during mid-gestation upon intranasal sow inoculation

4.1.1 Fetal PRRSV infection during mid-gestation upon intrafetal/intra-amniotic inoculation

4.1 Fetal PRRSV infection during late gestation

4.1.1 Fetal PRRSV infection during late gestation upon intranasal sow inoculation

4.1.1 Fetal PRRSV infection during late gestation upon intra-amniotic inoculation

4.1 Exploring endometrial/placental PRRSV infection

4.1.1 Why is PRRSV passage from mother to fetus restricted to late gestation?

4.1.1 PRRSV replication in the endometrium and placenta

4.1.1 PRRSV transmission from mother to fetus and from fetus to fetus

4.1.1 Cellular events in the maternal-fetal interface upon PRRSV infection

4.1.1 Pathological outcome of PRRSV infection in the maternal-fetal interface

5. Prevention of PRRSV infection in pregnant sows

6. Conclusions

7. Competing interests

8. Authors’ contributions

9. Acknowledgements

10. References

## 1. Morphology and function of the porcine placenta

In order to get a full understanding of PRRSV-induced reproductive failure, we will first review morphology and function of the porcine placenta.

Gestation begins with fertilization of an ovulated oocyte by sperm. After fertilization, the zygote undergoes time-dependent mitotic divisions, resulting in different cleavage stage embryos. Pig embryos reach the uterus on days 2–3 and migrate as blastocysts through both uterine horns between days 6 and 12 after ovulation [1]. In the uterus, blastocysts attach to the uterine epithelial cells at 13–14 days after fertilization [2]. Implantation involves phases of trophoblast-uterine epithelial cell apposition, adhesion, and microvillus attachment [3]. The initial implantation is accomplished primarily by the omphalochorion (yolk sac covered by trophoblast), which is then the dominant membrane, albeit only for a short time. On day 14, the allantois develops, and placental development starts 17 days after fertilization. By day 24–30 of gestation, the allantois attaches all around the periphery, the yolk sac shrinks and the placenta is completely established [4].

Mossman defines the placenta as an intimate apposition or fusion of fetal organs to maternal tissues for physiological interchange [4]. The placenta is an essential organ in permitting viviparity, a reproductive strategy acquired by eutherian mammals, in which fetal development proceeds within the female reproductive tract. Thus, placentation is fundamental in creating the environment in which the embryo and fetus develop. The quality of the embryonic and fetal environment has long lasting effects, influencing postnatal health and disease [5].

**Figure 1:**
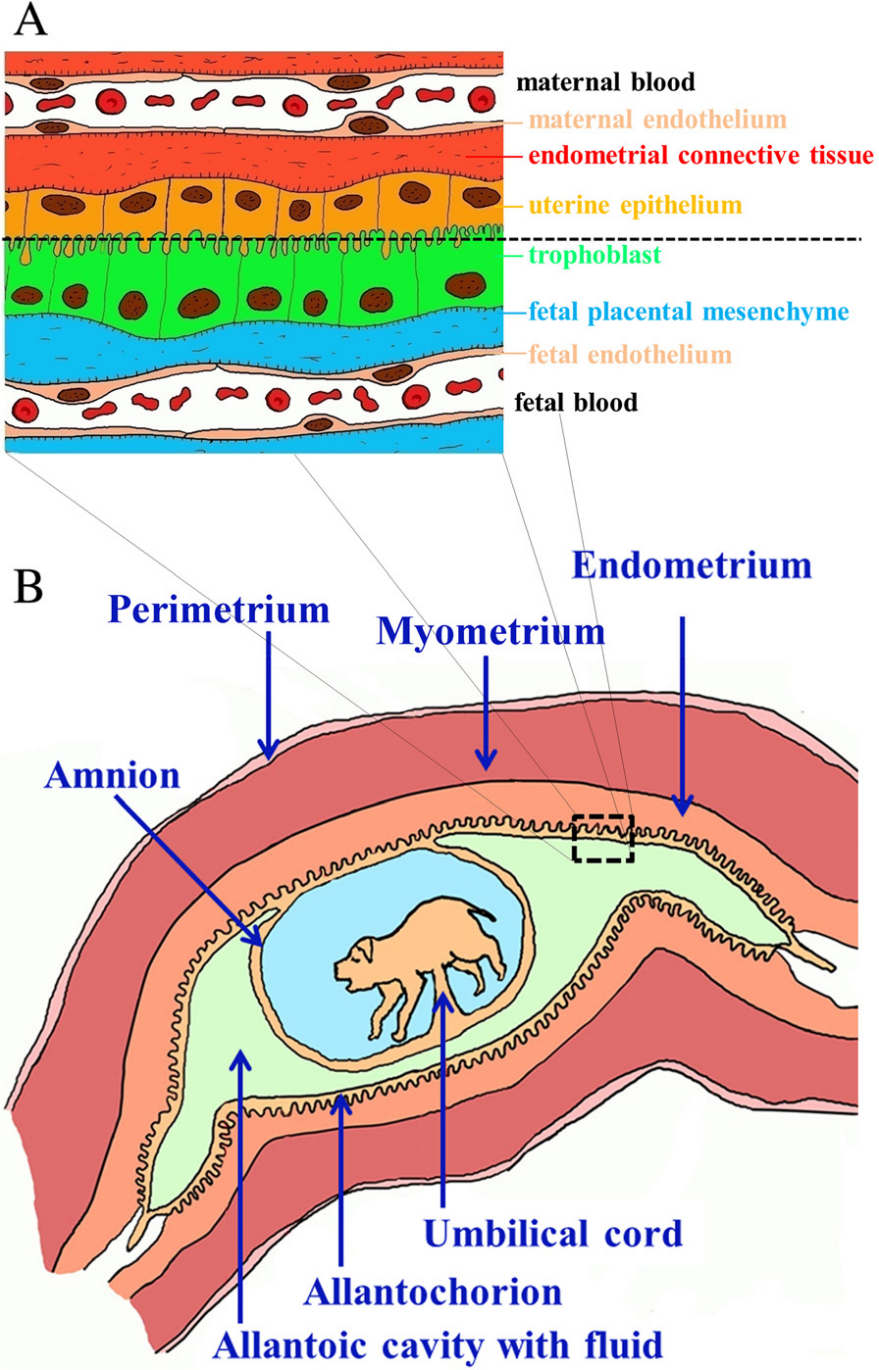
**Porcine placental barrier and conceptus.** Representations of the placental barrier in swine **(A)** and conceptus within the uterine horn **(B)**.

Based on histology, the placentae of eutherians are currently grouped in epitheliochorial, synepitheliochorial, endotheliochorial and haemochorial placentae. Pigs have an incomplete diffuse epitheliochorial placenta with atrophy at the peripheral tips. The swine placenta is a typical diffuse epitheliochorial organ without invasion [4]. Neither invasion of fetal tissue into the maternal endometrium, nor endometrial decidualization occurs. Instead, maternal and fetal microvilli appose and interdigitate giving a clear distinction between maternal and fetal tissues (semiplacenta). Therefore, in the present paper the terms “endometrium” and “placenta” are used to designate the maternal and fetal counterparts, respectively. Maternal and fetal blood is separated by six tissue layers (Figure 1A), which form a firm barrier. Even maternal antibodies are prevented to pass to fetuses during gestation [6]. Since no invasion occurs, much of the placenta/embryonic development depends on the uterine milk or embryotroph (endometrial gland secretions). Nevertheless, it is interesting to know that although the ungulate placenta is epitheliochorial, the placental barrier in certain regions is thinner than that found in carnivores with the endotheliochorial placenta (in this type of placenta, the endometrial epithelium under the placenta does not survive implantation, and fetal chorionic epithelial cells come in contact with maternal endothelial cells) [7].

The porcine placenta is diffuse, and almost the entire surface of the allantochorion (embryonic membrane consisting of a fused allantois and chorion) is involved in the formation of placenta (Figure 1B). The porcine chorion is formed by an extraembryonic mesoderm and trophoblast. Porcine fetuses have individual fetal membranes and starting from 39–55 days of gestation large placental zones of the individual concepti are terminated by the two extremities of the fetal sacs that include paraplacental and ischemic zones (necrotic tips) [8].

Pigs (and other mammals) have developed strategies that inhibit maternal immune responses to fetal alloantigens from paternal origin [9]. Thanks to this tolerance phenomenon, embryos and fetuses are not rejected. However, the mother must still be able to mount an effective defense against pathogens without rejecting the fetus. This is a complex task in the maternal-fetal interface, as its abundant blood supply makes it a favorable place for pathogens to prosper. A number of cell types within the maternal-fetal interface interact to protect concepti from rejection and suppress a germ infestation.

Trophoblast cells are specialized epithelial cells that possess several important functions [10]. Trophoblast cells play a key role in protecting the embryo/fetus from aggressive substances and preventing maternal immune rejection, ensuring appropriate bidirectional nutrient/waste flow required for growth and maturation of the embryo. Interestingly, the tolerance to fetal antigens occurs in the presence of a large number of maternal endometrial leukocytes [11]. Following placentation, distinct cellular changes in the local immune cell environment of the uterus are observed despite the non-invasive nature of the pig placenta. This happens presumably in response to trophoblast and/or fetal antigens. During the first month of gestation, at sites of conceptus attachment, the number of uterine lymphocytes decreases in the uterine epithelium and increases in the endometrial stroma. The majority of these lymphocytes express CD2 and CD8 surface markers, consistent with either T or natural killer (NK) cell lineage [9]. The role of T-lymphocyte subpopulations during the establishment of the epitheliochorial porcine placenta is largely unclear [3].

According to Haverson et al. [12], subpopulations of myeloid cells (neutrophils, eosinophils, monocytes and macrophages) in swine peripheral blood and lymphoid tissue express the SWC3 antigen. SWC3-expressing cells (most probably macrophages) are also commonly found in all layers of the endometrium of non-pregnant sows [13]; however the function of these cells has not yet been well studied. In the human decidua, macrophages express molecules that down-regulate inflammation and promote tissue remodeling as well as tolerance to foreign fetal (paternal) antigens [14].

## 2. Pathology of gestation in the swine

In the present section, a short overview [15,16] of reproductive pathology during porcine gestation is given.

Clinical manifestation of pathological processes depends on the period of prenatal development during which these processes occur. Death of embryos prior to implantation generally results in resorption of embryos and regular return to estrus in sows. Concepti are most probably also resorbed when death occurs between 14 and 35 days of gestation prior to skeleton formation. Sows will have an irregular return to estrus if all concepti die during that period or will farrow a small litter when only a proportion of the embryos die. Death of the fetus can be followed by mummification when it occurs after the embryonic period. Mummies are fetuses that died in utero and become exsiccated. Abortion results from the termination of gestation, with subsequent expulsion of all concepti and is associated with maternal and/or embryonic/fetal failure. Abortion takes place from day 14 (although abortion generally takes place during the fetal period starting from 25 days of gestation) to day 109 of gestation (fetuses expelled before day 109 of gestation normally cannot survive). Aborted sows return to estrus within 5–10 days or experience a prolonged anestrus. In abortion due to maternal failure, fetuses are generally all of the same size. A combination of mummies and dead piglets of variable size is observed when fetuses die at different times of gestation. Early farrowing occurs between 109 and 111 days of gestation and is associated with a high proportion of dead and low viable piglets. Stillbirth results from the expulsion of a dead fetus at term. Stillborn pigs die either prepartum or intrapartum and are grossly normal at birth.

## 3. PRRSV infection in pregnant sows

### 3.1. Introduction

In 1987, a new disease of unknown etiology characterized by reproductive failure in gilts and sows, and respiratory problems in young pigs, was observed in the USA and Canada [17,18]. Three years later, a similar outbreak was reported in Germany [19], with subsequent rapid spread through the major swine-producing areas in Western Europe. For the first time, a novel RNA virus was isolated from diseased animals and Koch’s postulates were fulfilled in 1991, in The Netherlands [20,21]. The etiological agent of PRRS was named Lelystad virus. Shortly thereafter, the etiology of PRRS was further confirmed by isolation of the virus and experimental reproduction of the disease in the USA [22]. In 1992, participants of the International Symposium on SIRS in Minneapolis agreed to name the causative agent porcine reproductive and respiratory syndrome virus (PRRSV). In the following years the virus was found not only in Western Europe and the USA, but also in Eastern Europe and some Asian countries [23-25]. At present, PRRS is the most economically important viral disease in swine livestock worldwide with an estimated annual cost of 560 million dollars in the USA [26]. In 2006, a highly pathogenic PRRSV infected more than 2 000 000 pigs in China and posed a great concern to the global swine industry [27].

### 3.2 Clinical signs

Clinical signs in pregnant gilts and sows during PRRSV outbreaks are mostly characterized by red/blue discoloration of the ears and vulva, late-term abortion storms, early farrowing and birth of mixed litters with living, stillborn fetuses and fetuses in different stages of mummification [20,23,28,29]. Clinical signs during endemic PRRSV infection vary from none to fever, lethargy, anorexia, pneumonia, regular and delayed return to estrus, sporadic late-term abortions, early farrowing and birth of mixed litters with living, stillborn fetuses and fetuses in different stages of mummification [20,23,28]. A fraction of animals within an affected livestock may escape from initial infection and serve as a susceptible target for subsequent waves of infection, supporting prolonged endemics [30]. PRRSV infection and associated clinical signs have been reproduced in experimental conditions. First experimental reproduction of congenital PRRSV infection in pregnant sows has been done by Terpstra et al. [20]. Shortly afterwards, research groups from all over Europe and the USA confirmed the etiological role of PRRSV in reproductive disorders [31].

### 3.3 Routes of PRRSV transmission

PRRSV can be transmitted horizontally following contacts between infected and naïve animals, as well as via semen of infected boars [32,33]. The duration of virus shedding in the semen of experimentally infected boars varies largely from 2 to 92 days after infection [34]. The virus can be shed with semen, even in the absence of viremia and in the presence of neutralizing antibodies [34]. This virus most likely reaches the tissues of the male reproductive tract and semen by migration of infected macrophages [35]. Once contaminated semen appears in the uterus, infection presumably starts from the endometrial tissues and regional lymph nodes following hematogenic or lymphogenic PRRSV dissemination throughout the sow organism and viremia appears [35].

## 4. PRRSV infection in the conceptus

Most previous investigations are mainly focused on transplacental PRRSV infection in pregnant sows and explored virus replication in fetal internal organs. Upon fetal infection, PRRSV replicates within fetal lymphoid tissues [29,36]. The fetal thymus, tonsils, and lymph nodes are the most regular sites for PRRSV replication [29,36,37]. However, the virus is also detectible in fetal lungs, liver, spleen, heart and kidneys [29,36-38]. The virus recovery from fetal tissues shows transplacental infection, but the absence of severe microscopic lesions in the internal organs of aborted or stillborn fetuses leaves the mechanism of reproductive failure unexplained [29,39-41]. The latter assumes that fetal death might be attributed to the virus-induced uterine/placental lesions. In accordance with this, transmission electron microscopy revealed virus-like particles in the endometrium and placenta collected from sows infected with PRRSV [42]. In several studies myometritis, endometritis, placentitis and microseparations in the maternal-fetal unit were described in samples from experimentally and naturally infected sows [31,39,42]. However, accurate quantification of virus-positive cells, their fate and virus colocalization with lesions in the endometrium and fetal membranes were not performed up till recently. In recent studies, new insight into the pathogenesis of PRRSV infection in pregnant sows was obtained by studying the virus replication and virus-induced pathology in the endometrium and placenta [37,43-45]. The main conclusion obtained from these studies is that PRRSV replication in the endometrium and placenta can contribute or even be a prerequisite to fetal death and reproductive disorders. The following subchapters highlight previous studies on transplacental and embryo/fetal PRRSV infection and our own exploration of endometrial/placental PRRSV infection.

### 4.1 Embryo PRRSV infection during early gestation

A reduction of swine reproductive performance, such as low conception and fertilization rate, during field PRRSV outbreaks might be attributed to PRRSV infection in the early stage of gestation [46]. As a result, experimental studies were performed to find out if exposure of gilts to PRRSV in the onset and early gestation can influence the conception rate and affect early embryos.

#### 4.1.1 Embryo PRRSV infection during early gestation upon intranasal sow inoculation

Inoculation of gilts at the day of insemination and sampling after 10 days results in fewer embryos than can be anticipated from the number of corpora lutea [47]. Later euthanasia of inoculated gilts (20 days after exposure) results in three times more dead embryos than in control non-inoculated gilts. Inoculation of gilts at 14 days of gestation also leads to embryonic infection (collection of embryos was performed at 20–22 days after maternal exposure to PRRSV) [48]; however, the incidence of embryonic infections is low, and the infected embryos do not show pathology. The susceptibility of embryos to PRRSV infection is also age-dependent. Ten-day-old embryos are not susceptible to PRRSV upon intranasal inoculation of the mother. Twenty-day-old embryos may contain infectious virus. It has been shown in vitro that preimplantation embryos are resistant to PRRSV infection [49,50]. The most probable explanation for this observation is the absence of PRRSV receptor sialoadhesin (Sn), on those embryos [50].

#### 4.1.2 Embryo PRRSV infection during early gestation upon in utero inoculation

Reports on outcomes of a sow insemination with PRRSV-contaminated semen [51] and in utero exposure of sows to the virus shortly after they had been bred naturally [52] also exist, but the results are contradictory. In the study of Prieto et al. [51], euthanasia of the in utero infected gilts at 20 days of gestation revealed the presence of dead and infected embryos. In contrast, in the study of Lager et al. [52] an effect of PRRSV exposure on the reproductive performance could not be demonstrated. Inoculated gilts that were euthanized at term or allowed to farrow showed no evidence of fetal infection, and the number of live fetuses or newborn piglets did not significantly differ from the control group. Remarkably however, in both studies PRRSV was transmitted from the lumen of the uterus to the blood circulation and internal organs of the dam [51,52].

Altogether, the described results suggest that PRRSV infection has little or no effects on conception rates despite the route of inoculation. However, it can result in death and infection of embryos after implantation.

### 4.2 Fetal PRRSV infection during mid-gestation

Only sporadic cases of mid-gestation abortions or small mummies at farrowing, indications of mid-term fetal infection, have been reported during PRRS outbreaks [53]. Despite this, some investigations were aimed at studying PRRSV infection in mid-gestation sows and fetuses upon experimental inoculation.

#### 4.2.1 Fetal PRRSV infection during mid-gestation upon intranasal sow inoculation

Virus inoculation of sows between 40 and 50 days of gestation does not cause pathology or PRRSV infection in fetuses [38,53]. Only in the study of Christianson et al. [53] the virus was isolated from few newborn pigs of sows inoculated in mid-gestation.

#### 4.2.2 Fetal PRRSV infection during mid-gestation upon intrafetal/intra-amniotic inoculation

Mid-gestation sows are susceptible to infection when exposed to the virus intranasally. However, transplacental infection is not reproducible during this period of gestation. Intrafetal or intraamniotic inoculation with PRRSV between 45 and 50 days of gestation results in productive infection in fetuses (sows were euthanized at 4–11 days after inoculation) [53], proving that the virus does not readily cross from mother to fetus upon maternal exposure in mid-gestation. In addition, the virus is not able to pass from the fetus to the mother in that period of gestation either [53].

As a conclusion, PRRSV replicates in mid-gestation porcine fetuses upon direct inoculation, but does not readily cross transplacentally from the mother to fetus when sows are exposed intranasally and from fetus to mother upon intrafetal/intra-amniotic inoculation.

### 4.3 Fetal PRRSV infection during late gestation

Clinical manifestation of PRRS in gilts and sows is mostly described as late-term reproductive failure. A number of studies were aimed at providing the experimental evidence of transplacental PRRSV infection and reproductive failure in late-gestation.

#### 4.3.1 Fetal PRRSV infection during late gestation upon intranasal sow inoculation

Experimental inoculation of sows with various PRRSV strains during late-gestation (72 to 93 days of gestation) consistently results in transplacental infection and reproductive failure that is similar to field observations [20,29,31,38,54,55]. A higher incidence of congenital infections and a higher number of infected fetuses/new-born pigs are observed upon inoculation of sows at 85–92 days of gestation in comparison to sows inoculated at 72 days of gestation [29,38]. In accordance with this, more pronounced reproductive disturbances are observed in sows inoculated later in gestation [38].

#### 4.3.2 Fetal PRRSV infection during late gestation upon intra-amniotic inoculation

Fetuses are susceptible to PRRSV infection upon intra-amniotic inoculation during late gestation [56]. Fetal inoculation during late gestation may result in fetal death within days after exposure.

### 4.4 Exploring endometrial/placental PRRSV infection

Recent studies indicate that the endometrium and placenta are involved in the PRRSV passage from the mother to fetus and that virus replication in the endometrial/placental tissues during late gestation is the actual reason for fetal death [37,43-45,57]. The following sections review the findings.

#### 4.4.1 Why is PRRSV passage from mother to fetus restricted to late gestation?

Despite the clear susceptibility of fetuses to PRRSV upon direct intra-fetal inoculation at any stage of gestation, exposure of sows and gilts to PRRSV only results in congenital infection and reproductive failure in late gestation in the field as well as under experimental conditions. Altogether, these observations indicate that the endometrium/placenta determines the passage of PRRSV to the embryos/fetuses. It is already known that PRRSV has a restricted tropism to Sn^+^CD163^+^ macrophages and that preimplantation embryos that do not have Sn^+^ cells cannot be infected [50,58]. Consequently, the presence of PRRSV target cells in the endometrium and placenta may be essential for virus passage from mother to fetus. Therefore, Sn^+^ and CD163^+^ macrophages, the cells potentially susceptible to PRRSV, were localized and quantified in the endometrium/placenta and organs of embryos/fetuses at different days of gestation (20–35, 50–60, 70–80, 114) [43]. A high number of CD163^+^ cells was observed within the endometrium/placenta during the entire period of gestation. Endometrial tissue samples collected at different stages of gestation also constantly contained Sn^+^ cells. In contrast, variations were observed in the placenta. At 50–60 days of gestation, Sn^+^ cells were not detected in the placenta of eleven fetuses out of fifteen tested. Almost all fetuses had Sn^+^ cells in the placenta at 70–80 days of gestation, and all fetuses had Sn^+^ cells at 20–35 and 114 days of gestation. The actual number of Sn^+^ cells in the placenta was considerably lower at 20–35 days of gestation (range 1–20; mean 7 cells/microscopic field) and even at 70–80 days of gestation (range 0–20; mean 6 cells/microscopic field) than at 114 days of gestation (range 3–48; mean 16 cells/microscopic field). In line with this, the high number of CD163^+^ and Sn^+^ cells was also observed in the endometrium and placenta collected at 100 and 110 days of gestation (data are not published). The abundance of cells that are highly susceptible to the virus in the placenta in late gestation may in part explain why congenital infection of PRRSV is mostly restricted to the end of gestation.

A previous challenge experiment revealed that the endometrial environment may also play an important role in the establishment of placental and transplacental PRRSV infections [37]. PRRSV-positive cells were not observed in the endometrial tissues adjacent to eleven fetuses of one sow that was intranasally inoculated with PRRSV at 70 days of gestation and sampled at 80 days of gestation, despite maternal viremia and the presence of endometrial CD163^+^ and Sn^+^ cells. In contrast, PRRSV efficiently replicated in the endometrium/placenta collected from sows intranasally inoculated with PRRSV at 90 days of gestation and sampled at 100 days of gestation. Two possible obstructions may be present for PRRSV to find its way from maternal blood to the endometrium before 90 days of gestation. Most probably PRRSV passage from maternal blood to endometrial connective tissues happens in association with blood monocytes migrating through endometrial vessels. Endometrial blood vessels might restrain cell-associated PRRSV passage from maternal blood to endometrial connective tissues at 70–80 days of gestation. Alternatively, yet unknown cellular/molecular endometrial factors may block PRRSV replication in local macrophages (in addition, virus-infected macrophages may be rapidly eliminated) at 70–80 days of gestation. A combination of these scenarios is also possible. The comparative studies of the endometrium from healthy and PRRSV-infected sows at different terms of gestation may lead to an understanding of those factors, and as a consequence, may help in designing new antiviral strategies to prevent placental and transplacental infections.

Altogether, the still unknown factors that prevent or block PRRSV replication in the endometrium and the lack of susceptible cells in the placenta might join forces and cause resistance to placental/transplacental PRRSV infection before 90 days of gestation. Afterwards, the endometrium and fetal membranes become more susceptible to PRRSV which most probably contributes to the late term appearance of placental/transplacental PRRSV infection and associated reproductive problems.

#### 4.4.2 PRRSV replication in the endometrium and placenta in late gestation

PRRSV-target cells are present in the endometrium/placenta and PRRSV-infected macrophages in other organs die by apoptosis. However, accurate quantification of virus-positive cells, their fate and virus colocalization with lesions in the endometrium and fetal membranes were not performed until recently. In a recent study, PRRSV-positive and apoptotic cells were identified, localized and quantified in the endometrium/placenta from three sows inoculated at 90 days of gestation and euthanized 10 days later [37]. As a control, non-inoculated sows were included in the study. At 10 days-post inoculation, challenged sows were viremic and PRRSV spread from mother to fetus was detected in all of them. Severe histopathological lesions were not observed in the endometrium/fetal placenta collected from inoculated and control animals. In inoculated sows, PRRSV replication was detected in the endometrium and placenta via a specific immunofluorescence staining. The number of PRRSV-positive cells in the placenta (1-289/10 mm^2^ of tissue) was significantly higher than in the endometrium (1-16/10 mm^2^ of tissue; *p* = 0.004). The amount of apoptotic cells was significantly higher in the PRRSV-positive endometrium from inoculated sows versus virus-negative tissues from control sows. The amount of apoptotic cells increased significantly in the PRRSV-positive placentae compared to the PRRSV-negative placentae. In the placenta a spatial correlation between the sites of the PRRSV replication and apoptotic cells was observed. The main conclusion obtained from the study is that PRRSV replicates in the endometrium/placenta and causes apoptosis of local cells in late gestation.

#### 4.4.3 PRRSV transmission from mother to fetus and from fetus to fetus

**Figure 2:**
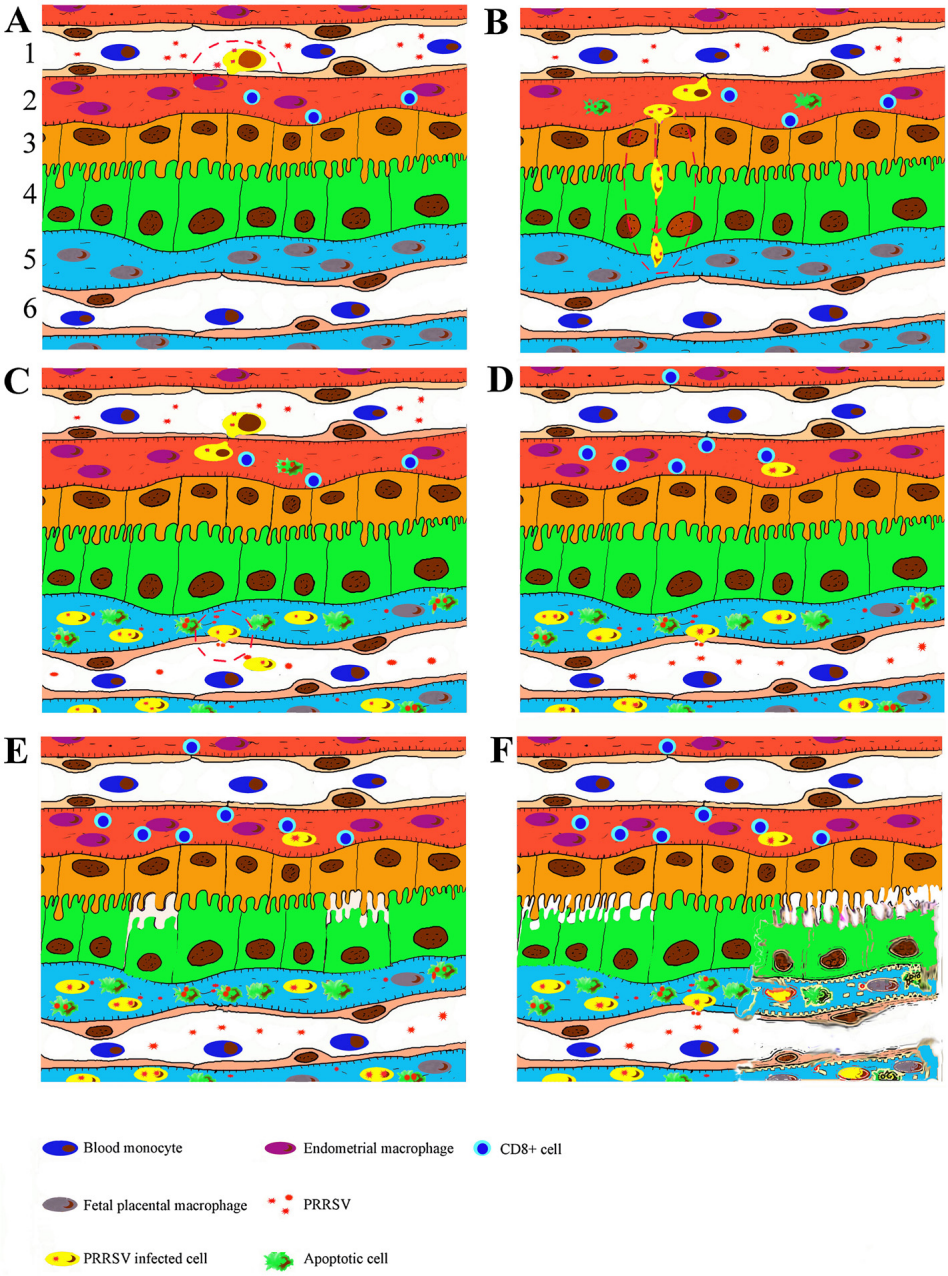
**Events during PRRSV infection in the maternal-fetal interface in sows intranasally inoculated at 90 days of gestation.** 1. Maternal blood vessel. 2. Endometrial connective tissue. 3. Uterine epithelium. 4. Trophoblast. 5. Fetal placental mesenchyme. 6. Fetal blood vessel. **(A)** During viremia PRRSV attaches and probably enters and replicates in susceptible monocytes adhering to the endothelial cells of the endometrial vessels. Extravasation of the PRRSV-bearing monocytes from maternal blood to the endometrium. **(B)** PRRSV replicates in the endometrial macrophages. PRRSV causes apoptosis in infected and surrounding cells during replication within the endometrium. PRRSV crosses the uterine epithelium and trophoblast, most probably in association with maternal macrophages. **(C)** Focal, highly efficient PRRSV replication in the fetal placental macrophages. PRRSV reaches fetal internal organs most likely through the umbilical circulation. PRRSV causes apoptosis in infected and surrounding cells during replication in the placenta. **(D)** Maternal immunity (most probably CD8^+^ endometrial NK cells) suppresses PRRSV replication within the endometrium. Focal, highly efficient PRRSV replication in the placenta. **(E)** Focal detachment of the trophoblast from the uterine epithelium and focal degeneration of the placenta, at the places of virus replication and probably in the adjacent sites. **(F)** Multifocal degeneration and finally full degeneration of the placenta, at the places of virus replication, and probably in the adjacent sites.

Maternal viremia leading to PRRSV replication in the endometrium with subsequent fetal infection through the placenta is the most probable way of PRRSV transmission from mother to fetus. The hypothetical model of events during PRRSV infection in the endometrium/placenta is given in Figure 2.

Similar to young pigs, upon intranasal inoculation of sows, the primary sites for PRRSV replication are most probably the respiratory tract and tonsils. Afterwards, viremia occurs and short-term viremia is sufficient to deliver PRRSV into the endometrial vessels. It has been shown that in guinea pigs and rats, blood monocytes pass through blood vessel walls via diapedesis into the tissues to further differentiate into macrophages (extravasation). Diapedesis of monocytes can happen by migration straight through the microvascular monolayer of endothelial cells (the transcellular route) or in between the endothelial cells (the paracellular route) [59,60]. Diapedesis begins with the accumulation of leukocytes on the luminal surface of the endothelium through a sequence of rolling, activation, and firm adhesion [60]. A variety of monocyte and endothelium molecules and signaling pathways are involved in trafficking [61,62]. Most probably blood monocytes in pigs, like in guinea pigs and rats, pass through endometrial vessel walls via diapedesis into the endometrial connective tissues to further differentiate into macrophages. Prior to or during diapedesis (when blood monocytes differentiate) blood monocytes may obtain the ability to attach PRRSV and probably become infected. Upon invasion the virus enters the endometrium with (on/in) differentiated monocytes (Figure 2A). In support of this scenario, PRRSV-positive cells are observed in between endothelial cells of endometrial blood vessels [37].

The next step after PRRSV replication in the endometrium towards placental infection is the virus crossing the uterine epithelium and trophoblast layers. There are three hypothetical modes of PRRSV crossing through the uterine epithelium-trophoblast barrier:

– Direct cell to cell spread of PRRSV from infected endometrial macrophages to the uterine epithelium and subsequently through the trophoblast cells to placental macrophages.

– Spread of free PRRSV particles directly through the uterine epithelium and trophoblast cells or between them.

– Cell-associated PRRSV spread from the endometrium to concepti in/on macrophages migrating from the mother to the fetus.

The direct spread of PRRSV from endometrial macrophages to the uterine epithelium and afterwards through the trophoblast to fetal placental macrophages is less probable than the two other ways of maternal-fetal virus transmission. PRRSV has a very restricted tropism to macrophages, and all PRRSV-infected cells within the maternal-fetal interface are macrophages positive for CD163 and Sn [37]. In contrast, the uterine epithelium and trophoblast do not express these molecules and are not susceptible to infection.

Pigs have an epitheliochorial placentation and even antibodies are hindered in passing from the dam to the fetus during porcine gestation. Taking into account that IgG antibodies (12 nm) are significantly smaller than the PRRSV virion (55 nm), the probability of the free PRRSV particle spreading through the uterine epithelium/trophoblast is very low. The ability of free virus particles to pass might also depend on the integrity of the placental tissue layers. Mechanical rupture of one or several tissue layers that separate maternal and fetal blood circulations may facilitate PRRSV transmission. In a few cases, during the opening of uterine walls, we observed hemorrhages under the alantochorion surface. However the majority of the infected and negative fetuses did not have any visible hemorrhages within the adjacent uterine wall. It is not clear if the observed hemorrhages were present prior to sow euthanasia or only appeared during euthanasia and if they play any significant role in placental and transplacental PRRSV infection.

**Figure 3:**
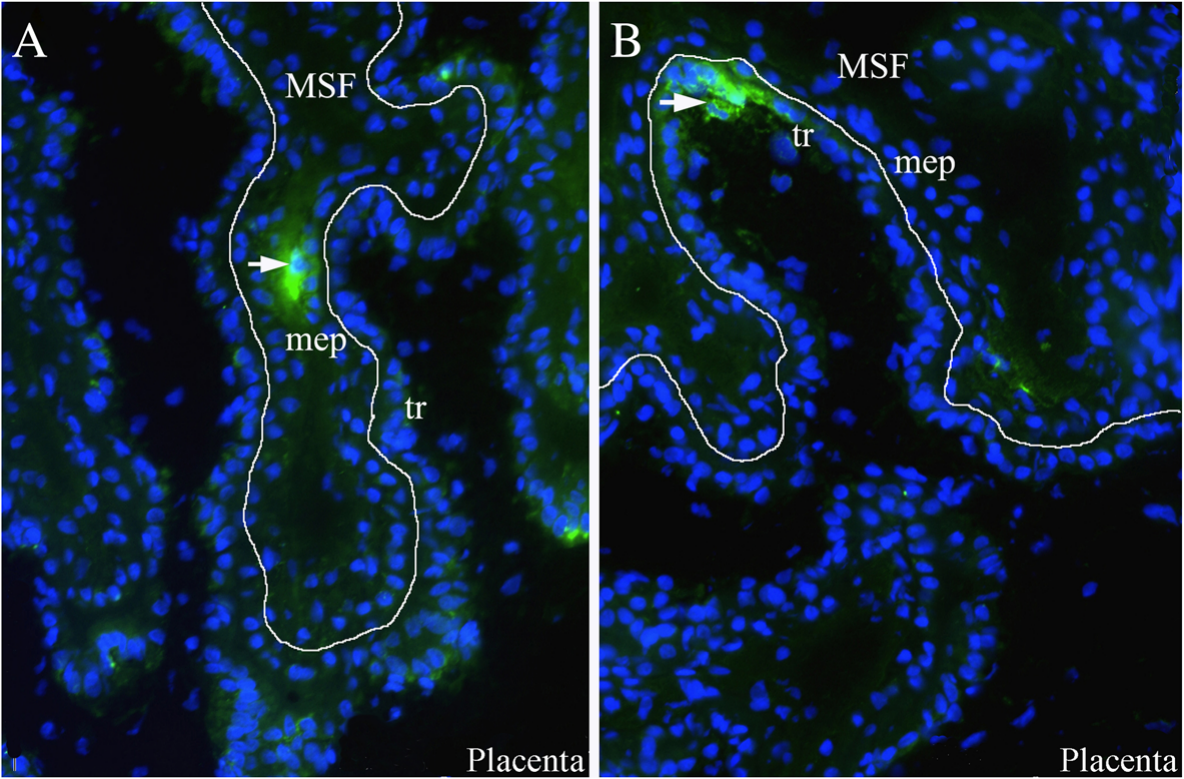
**PRRSV-positive macrophages in the endometrium (A) and placenta (B).** PRRSV-positive macrophages (arrowed) in close proximity to the uterine (maternal) epithelium (mep) and fetal trophoblast cells (tr). MSF: maternal secondary fold.

To the author’s opinion, cell-associated virus spread from the endometrium to the fetal membranes, in/on cells migrating from the mother to the fetus, is most plausible (Figure 2B). The involvement of cell trafficking from the mother to the fetus has been previously proposed for congenital HIV [63] and LDV [64] infection. In case of PRRSV infection, this hypothesis gains support from several lines of evidence. PRRSV has a very restricted tropism to some subsets of macrophages and all infected cells within the endometrium are CD163^+^ and Sn^+^. Hypothetically, PRRSV-infected macrophages may cross through the uterine epithelium and trophoblast layers. The trafficking of different cell types across the placenta is common in human and rodent pregnancies [65]. Humans have hemochorial placentation which displays major differences from porcine epitheliochorial placentation. Villi of the human placenta are covered by the syncytiotrophoblast (an outer layer, maternal side) and cytotrophoblast (an inner layer, fetal side). During the first trimester of gestation the villi have a nearly complete cytotrophoblast layer underneath the syncytiotrophoblast layer. In later pregnancy, the internal cytotrophoblast layer is discontinuous, and the syncytiotrophoblast layer is the only barrier to be overcome by maternal cells on their way to the fetal membranes. If transplacental cell migration occurs during porcine gestation and if it is responsible for PRRSV transmission from the mother to fetus, two additional tissue layers besides the trophoblast (syncytiotrophoblast in comparison to humans) have to be crossed by migrating cells: the endometrial connective tissues and the uterine epithelium. PRRSV-target and virus-positive cells are abundant within the endometrial connective tissues and these cells are possibly free to move. It is yet unknown if local endometrial cells can pass through the uterine epithelium and porcine trophoblast. During screening of uterine tissue sections stained with PRRSV-specific antibodies, occasional virus-positive cells are observed in extremely close proximity to the uterine epithelium and trophoblast (Figure 3). Those infected cells might be migrating through the uterine epithelium and trophoblast. Moreover, in our previous study, female cells were demonstrated within the male fetuses. Those cells might have a fetal, maternal or double origin [44]. The observations that the number of female microchimeric cells within male fetal tissues is significantly higher than the number of male microchimeric cells within female fetal tissues is in agreement with the scenario of a double origin (sibling and maternal) [44]. It can be proposed that maternal macrophages are able to migrate from the mother to fetus and serve as “Trojan horses” for PRRSV invasion. However, the origin (maternal, sibling or double) of female microchimeric cells within male fetuses, the mechanism of their transmigration and their ability to transfer infectious PRRSV remain to be experimentally studied and proven.

After passing the uterine epithelium and trophoblast, PRRSV-bearing cells reach the fetal placenta. Subsequently, a focal, very efficient PRRSV replication is observed (Figure 2C). During PRRSV replication in the fetal placental macrophages, virus-positive cells are localized close to the fetal placental blood vessels [37]. Afterwards, PRRSV-infected placental cells may reach fetal blood and organs. In accordance with this, reverse migration of inflammatory monocytes from tissues back to the vascular circulation has previously been described [66].

PRRSV replication is observed within the fetal liver, thymus, spleen, lungs, inguinal and mesenteric LN in late gestation [37,44,45]. This is in line with the findings that susceptible CD163^+^Sn^+^ macrophages are abundant within the fetal internal organs [43]. PRRSV is also detected in amniotic fluids (11 virus-positive amniotic fluids out of 34 tested, PRRSV titres 1.3-5.8 log10 TCID_50_/mL; PRRSV-positive cells were also detected in amniotic membranes of several fetuses; sows were inoculated at 90 days of gestation and sampled 20 days later) (unpublished data). During productive PRRSV replication within fetal membranes, viral passage from fetus to fetus may accelerate PRRSV infection of the fetuses in utero. PRRSV can be carried on/in migrating macrophages and/or as free virus between siblings via adherent extremities of fetal membranes or through blood anastomoses which may exist between allantochorions of neighboring concepti [8,67]. The use of carrier cells is indirectly supported by the existence of sibling microchimerism. Male cells are observed within the liver and lungs of female fetuses and vice versa [44]. The proposed way of virus in utero dissemination can be utilized by other porcine pathogens. For example, intrauterine spread of porcine parvovirus between fetuses has been demonstrated [68].

#### 4.4.4 Cellular events in the maternal-fetal interface upon PRRSV infection

After invasion into the endometrium, PRRSV replicates within susceptible local macrophages. During the course of infection, immunity slowly clears the virus within the endometrium (Figure 2D) [45]. As a result, less efficient PRRSV replication is observed in the endometrium of sows euthanized at 20 days post-inoculation (range 0–4, mean 0.2 cells/10 mm^2^) [50] than in the endometrium of sows euthanized at 10 days post inoculation (range 0–16, mean 4 cells/10 mm^2^) [37]. The depletion of susceptible Sn^+^ and CD163^+^ cells was not observed in these samples (data are not published). The exact nature of this local antiviral immunity within the endometrium is not clear. Interestingly, sows inoculated with PRRSV at 90 days of gestation and sampled 10 days later have a higher number of Sn^+^ cells in the endometrium and placenta due to de novo Sn expression on local CD163^+^ cells [57]. Along with the increased number of Sn^+^ macrophages an increased number of CD8^+^ cells, which are mostly CD3^-^ and previously described as uterine NK cells [69-71], is observed in the PRRSV-positive endometrium. It is possible that endometrial CD8^+^ cells activated by Sn^+^ macrophages take part in suppression of PRRSV replication [57]. Therefore, the absence or small numbers of CD8^+^ cells in the fetal allantochorion may be a reason for the very efficient PRRSV replication (in comparison to the endometrium) [57]. Macrophages infected with PRRSV possess a reduced susceptibility toward peripheral blood NK cytotoxicity in vitro [72]. However, endometrial NK cells are unique and distinct from those from the peripheral blood due to the special immunological environment in the maternal-fetal interface [73,74]. For example, uterine NK cells can be induced to secrete soluble factors that can inhibit HIV infection in vitro [75]; in contrast, human peripheral blood NK cells do not exhibit such activity.

The excessive number and/or altered function of endometrial/placental macrophages can theoretically disrupt a delicate immunological balance in the maternal-fetal interface and contribute to the previously described placental lesions [45]. Upon PRRSV infection, differentiated Sn-positive macrophages might activate endometrial NK cells; afterwards, activated NK cells might damage the semiallogeneic trophoblast [57]. The capacity of porcine endometrial NK cells to kill semiallogeneic trophoblast cells is already known [76]. Consistent with this notion, the number of CD8^+^ cells in close proximity to the uterine epithelium is significantly higher in PRRSV-positive samples versus PRRSV-negative samples [57].

More comprehensive understanding of local defensive mechanisms in the endometrium and placenta upon PRRSV infections can help to design new anti-viral strategies which may be able to block virus replication prior to an establishment of placental/transplacental infection. Moreover, this knowledge will extend a general insight into anti-microbial immunity during gestation.

#### 4.4.5 Pathological outcome of PRRSV infection in the maternal-fetal interface

Taking into account the highly efficient PRRSV replication within the fetal placental mesenchyme and the crucial role of the placenta for the normal development of fetuses during gestation, it is plausible that virus-induced placental damages are responsible for PRRSV-related reproductive problems. At 20 days post-inoculation (inoculation at 90 days of gestation), severe histopathological lesions, which range from local separation between the uterine epithelium and trophoblast to complete degradation of the fetal placental mesenchyme, are observed (Figure 2E and F) [45]. These histopathological lesions are incompatible with fetal life, since the integrity between the maternal and fetal counterparts within the maternal-fetal interface is crucial for in utero gas (O_2_/CO_2_) exchange, feeding and clearing of toxic metabolites of the progeny. On the one hand, these lesions can be initiated by PRRSV damage of susceptible fetal placental macrophages and bystander cells. At 10 days post-inoculation, a significantly higher number of apoptotic (and probably necrotic) cells is observed within the fetal placental mesenchyme from the PRRSV-inoculated sows compared to the non-inoculated control sows [37]. This massive killing of cells may lead to destruction of the fetal placental mesenchyme, which supports the trophoblast layer. On the other hand, PRRSV replication in endometrial/placental macrophages can indirectly influence the expression of integrins and extracellular matrix proteins (or other junction proteins) on the trophoblast and/or uterine epithelium. These proteins are important for successful implantation and placentation in pigs [77-80]. As a result, separations between the uterine epithelium and trophoblast may appear. These separations have been previously detected via electron microscopy [42] and were also observed in our recent study [45]. Subsequently, deterioration of placental functions and finally placental degradation lead to fetal death and clinical representations of congenital PRRSV infection. In a recent study, PRRSV-positive cells were also localized in the myometrium [45]. PRRSV infection may influence and derange the timely myometrial quiescence and activation which are important during gestation and parturition, respectively.

To cause late abortion or preterm birth, PRRSV probably induces severe lesions in the maternal-fetal interface of most if not all fetuses. If the virus replicates in the maternal-fetal interface adjacent to a limited number of fetuses, gestation ends at term but the infection might result in stillbirth and/or the birth of living PRRSV-positive fetuses. Even in the case of fetal survival, the placental damages induced during the fetal period most probably have prolonged negative consequences. The quality of the embryonic and fetal environment has lasting effects, influencing postnatal health and disease [5].

## 5. Prevention of PRRSV infection in pregnant sows

Field reports suggest that following PRRSV-induced reproductive failure, sows develop a protective immunity. These observations are based on the fact that affected sows have a normal litter following rebreeding despite the apparent circulation of the virus within the herd [81]. Experimental data also prove full protection of swine to a homologous PRRSV challenge [54,82]. Therefore, vaccination is considered as the principal method to control and treat PRRSV infection [83,84].

Two types of PRRSV vaccines are available: modified live virus vaccines (MLV vaccines also called attenuated vaccines), and killed virus vaccines (KV vaccines also called inactivated vaccines). Attenuated vaccines are generated by in vitro cell culture passaging of virulent virus until an attenuated phenotype appears. Inactivated vaccines are generated by chemical or physical inactivation of virulent viruses. MLV and KV vaccines are developed both from European and North American PRRSV strains, and are used on most continents, since a strict geographical genotype barrier no longer exists. Commercial, inactivated vaccines are authorized for use in breeding pigs as they should provide protection without deleterious effects on reproduction. However, inactivated vaccines do not induce virus neutralizing antibodies and cannot prevent PRRSV infections, even after challenge with homologous strains [85-89]. In contrast, attenuated vaccines are able to induce virus neutralizing antibodies and prevent virus replication in target cells, viremia and clinical symptoms [90,91]. Despite these positive effects full protection is only achieved against homologous strains. Moreover, outbreaks of the acute syndrome, characterized by abortion and high mortality in pregnant sows, have been described after vaccination with the North American type MLV vaccines [91,92]. Live North American vaccine virus has also been isolated after field clinical cases of PRRS [93]. Live vaccine virus has an unwanted tendency to spread not only within the vaccinated herds but also to neighboring non-vaccinated herds. During spreading among pigs, the vaccine virus can revert genetically [94-96], which apparently leads to the observed clinical problems [93]. Experimental inoculation of sows with North American vaccine-derived PRRSV in late gestation results in subsequent transplacental infection in accordance with field observations [97]. European-type vaccine PRRSV can also replicate in gilts after intranasal exposure and even cross from the mother to the fetus, however with a less detrimental effect on the reproductive performance [98].

In a recent study, a killed PRRSV vaccine, produced using a new quality-controlled viral inactivation procedure and applied with a suitable adjuvant, was tested [45]. The results showed that the new inactivated vaccine is able to prime the VN antibody response and to slightly reduce the duration of viremia in gilts. It also reduces the number of PRRSV-positive fetuses, and improves fetal survival, but is not able to prevent congenital infection. Positive effects were most probably achieved via reduction of the virus transfer from the endometrium (the primary site for PRRSV replication prior to conceptus infection) to the placenta, because the number of PRRSV-positive cells in the placentae was significantly higher in unvaccinated versus vaccinated gilts [45]. This vaccine may be recommended for use in endemically infected farms alone or in combination with other vaccines to reduce losses due to PRRSV infection in pregnant sows. The aim is to activate the VN antibody response before 80 days of gestation, when sows become susceptible to placental/transplacental infection. Vaccination of gilts with a live vaccine before insemination or during the early stage of gestation with subsequent boosting with the new inactivated vaccine may offer new perspectives for the prevention of PRRSV-induced reproductive disorders [99].

The requirements for laboratory testing of PRRSV vaccines, prior to field vaccine evaluation in pregnant sows were also summarized [45]. This may be helpful in writing a monograph. Three specific requirements are crucial for PRRSV vaccine testing in pregnant sows. First of all, the challenge should be performed at the end of gestation (90 days of gestation), the time when pregnant sows are the most susceptible to congenital PRRSV infection. Secondly, a combination of different techniques must be used to detect PRRSV in the following maternal and fetal tissues: maternal blood, endometrium, fetal placenta, fetal blood, thymus and liver. Finally, examination of fetuses for gross pathology and of the endometrium/placenta for virus-induced microlesions is required for the evaluation of the candidate vaccine efficacy.

Alternatively, other immunotherapeutic strategies aiming at the complete block of virus replication within the endometrium, preventing PRRSV to be transmitted from the endometrium to concepti, should be considered. Blocking virus replication within the endometrium may be achieved by the manipulation of the local immune cells responsible for PRRSV elimination. Targeted delivery of immunotherapeutic agents via target cell receptor-specific immunoconjugates prior to infection may be a useful strategy. The candidate cells for such targeting in the endometrium might be CD8^+^ cells (presumably uterine NK cells, described above). Better insight into the immune determinants able to control maternal viremia, virus replication within the endometrium and virus transmission from the endometrium to the placenta, will finally lead to an appropriate preventive strategy.

## 6. Conclusions

In conclusion, the recent data strongly indicate that PRRSV replicates and causes pathology in the endometrium and placenta in late gestation. Virus replication in the endometrium and placenta are responsible for the range of PRRSV-related reproductive problems. More comprehensive understanding of defensive immune mechanisms in the endometrium and placenta upon PRRSV infection can help to design new immunotherapeutic strategies that may be able to block virus transmission from mother to conceptus. Moreover, this knowledge will extend general insight into anti-microbial immunity in the maternal-fetal interface.

## 7. Competing interests

The authors declare that they have no competing interests.

## 8. Authors’ contributions

UUK reviewed the literature, performed analysis and prepared the manuscript. HJN helped in writing the manuscript. Both authors read and approved the manuscript.
